# Pathways to Mental Health–Related Treatment Among Caregiving Spouses

**DOI:** 10.1093/geroni/igaa057.1151

**Published:** 2020-12-16

**Authors:** Hyojin Choi, Kristin Litzelman, Molly Maher, Autumn Harnish

**Affiliations:** 1 University of Wisconsin-Madison, Madison, Wisconsin, United States; 2 University of Wisconsin - Madison, Madison, Wisconsin, United States; 3 University of Wisconsin-Madison, Fitchburg, Wisconsin, United States

## Abstract

Spouses of cancer survivors are 33% less likely to receive guideline-concordant depression treatment than other married adults. However, depression is only one of many manifestations of psychological distress for caregivers. This exploratory study sought to assess the paths by which caregivers access mental health-related treatment. Using nationally representative data from the Medical Expenditures Panel Survey, we assessed the proportion of caregivers who received a mental health-related prescription or psychotherapy visit across care settings (office based versus outpatient hospital, emergency room, or inpatient visit), provider type (psychiatric, primary care, other specialty, or other), and visit purpose (regular checkup, diagnosis and treatment, follow-up, psychotherapy, other). In addition, we assessed the health condition(s) associated with the treatment. The findings indicate that a plurality of caregivers accessed mental health-related treatment through an office-based visit (90%) with a primary care provider (47%). A minority accessed this care through a psychologist or psychiatrist (11%) or a physician with another specialty (12%) or other provider types. Nearly a third accessed treatment as part of a regular check-up (32%). These patterns did not differ from the general population after controlling for sociodemographic characteristics. Interestingly, mental health-related treatments were associated with a mental health diagnosis in only a minority of caregivers. The findings confirm the importance of regular primary care as a door way to mental healthcare, and highlight the range of potential paths to care. Future research will examine the correlates of accessing care across path types.

